# A boy with amblyopia and familial exudative vitreoretinopathy harboring a new mutation of *LRP5* and *OPA1*: A case report

**DOI:** 10.3389/fgene.2022.998846

**Published:** 2022-09-29

**Authors:** Chunli Chen, Sitong Guo, Rui Zhao, Shoubin Liu, Jingjing Wu, Yuanyuan Xiao, Simeng Hou, Libin Jiang

**Affiliations:** ^1^ Beijing Tongren Eye Center, Beijing Tongren Hospital, Capital Medical University, Beijing Ophthalmology and Visual Sciences Key Laboratory, Beijing, China; ^2^ Beijing Institute of Ophthalmology, Beijing, China

**Keywords:** FEVR, new mutation of LRP5, OPA1, fraternal twin, case report

## Abstract

**Background:** The study aimed to report a boy with familial exudative vitreoretinopathy and amblyopia harboring a new mutation of the *LRP5* and *OPA1* gene abnormality.

**Case presentation**: A 9-year-old boy presented with a 2-year history of deteriorating visual acuity in the right eye. His best-corrected visual acuity was −7.00/−1.75 × 100 = 0.3 in the right eye and −2.50/−1.50 × 170 = 0.8 in the left eye. Two autosomal dominant gene mutation sites were identified in the patient: *LRP5* (c.2551C > T, *p*.His851Tyr) from his father and *OPA1* (c.565G > A, *p*.Glu189Lys) from his mother. Interestingly, his fraternal twin brother harbored no abnormal gene mutations, and his eye tests were normal.

**Conclusion:** This case expands the spectrum of *LRP5* gene mutations among Chinese patients with familial exudative vitreoretinopathy, and it is the first time to report a patient harboring both LRP5 and OPA1 gene mutations having anisometropic amblyopia and strabismus as the primary manifestations. These four family members exhibited individual heterogeneity of phenotypes and genotypes associated with hereditary ophthalmopathy. A comprehensive analysis of clinical phenotypes and genotypes provides clinical clues for improving the level of clinical and genetic diagnoses and a deeper understanding of the disease.

## Background

Familial exudative vitreoretinopathy (FEVR) is an inherited disorder of vitreoretinopathy usually observed in full-term infants. It is characterized by incomplete and abnormal vascularization of the peripheral retina, which can lead to the displacement of the macula secondary to traction, falciform folds of the temporal retina, retinal neovascularization, vitreous hemorrhage, tractional retinal detachment, and subretinal exudates. FEVR was first described by Criswick and Schepens (1969). According to sequencing data reported in the literature to date, 14 pathogenic genes have been found to be associated with FEVR, including *NDP, FZD4, LRP5, TSPAN12, ATOH7, ZNF408, KIF11, RCBTB1, CTNNB1, ILK, JAG1, CTNNA1, TGFBR2,* and *DLG1* (Zhu et al., 2021). Approximately one half of patients with FEVR harbor mutations in *NDP, FZD4, LRP5*, and *TSPAN12*, which encode components of the norrin/β-catenin pathway through inactivation of norrin/β-catenin signaling (Panagiotou et al., 2017; Li et al., 2018) and play critical roles in retinal angiogenesis by variable patterns of inheritance and penetrance. FEVR is a highly variable disease without distinct associated phenotypes or genotypes. Autosomal dominant optic atrophy (ADOA) is one of the most common types of autosomal dominant hereditary optic neuropathies, with an incidence of 1:10,000–1:50,000. ADOA varies within and between large families, with a penetrance rate of 40–90%, which is incomplete (Pretegiani et al., 2017). Approximately two-thirds of cases occur in the first decade of life (Ham et al., 2019). To date, only two pathogenic genes of ADOA have been identified: *OPA1*, located on chromosome 3q28-29, and *OPA3*, located on chromosome 19q13. Approximately 60%–80% of ADOA cases are linked to mutations in the *OPA1* gene (OMIM #165500) (Le Roux et al., 2019; Lenaers et al., 2021). We report a case involving a fraternal twin patient and his family with simultaneous mutation of *LRP5* and *OPA1* pedigrees. Varying clinical phenotypes and gene mutation types among different family members fully demonstrate the individual heterogeneity of phenotypes and genotypes related to hereditary ophthalmopathy.

## Case presentation

**FIGURE 1:**
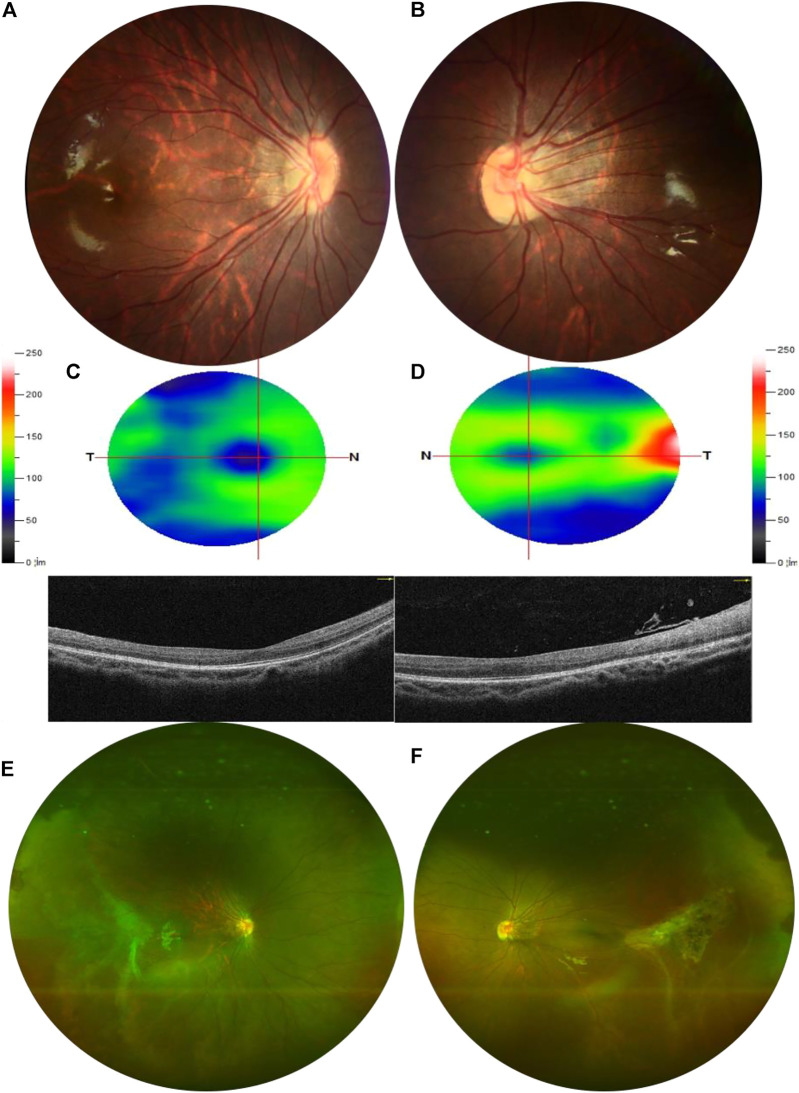
Examination results of the proband. **(A,B)** Color of the optic disc was slightly pale in both eyes, and the retinal blood vessels were stretched to the temporal side, which was obvious in the left. **(C,D)** OCT showed that the inner and outer layers of the central fovea in the right eye were normal but diminished foveal contour with persistent inner foveal retinal layers in both eyes and no foveal depression on the left. The thickness of the superior retinal nerve fiber layer (RNFL), inferior RNFL, and average RNFL was within the normal range. The thickness of the superior ganglion cell complex (GCC), inferior GCC, and average GCC was within the normal range. **(E,F)** Peripheral retinal blood vessels were flat and numerous with avascular areas in both eyes. The peripheral retina of the left eye was accompanied by mild stretch proliferation and pigmentation changes.

**FIGURE 2:**
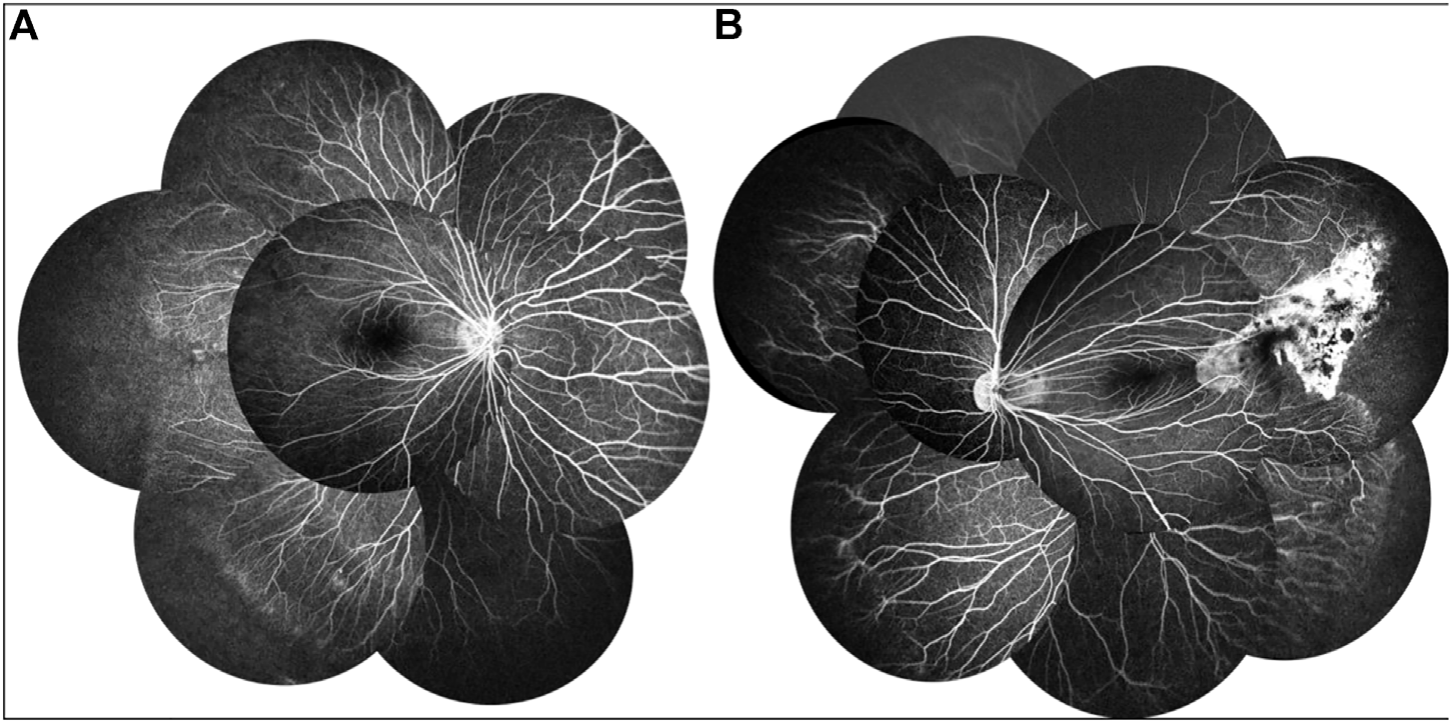
FFA of the proband showed a large avascular zone in the bilateral temporal peripheral retina, blood vessels in the peripheral retinal were flat and numerous, and the optic disc showed high fluorescence in the late stage of angiography **(A,B)**. The temporal peripheral retina of the left eye showed patchy high fluorescence with fluorescein staining, but no obvious leakage **(B)**.

**FIGURE 3:**
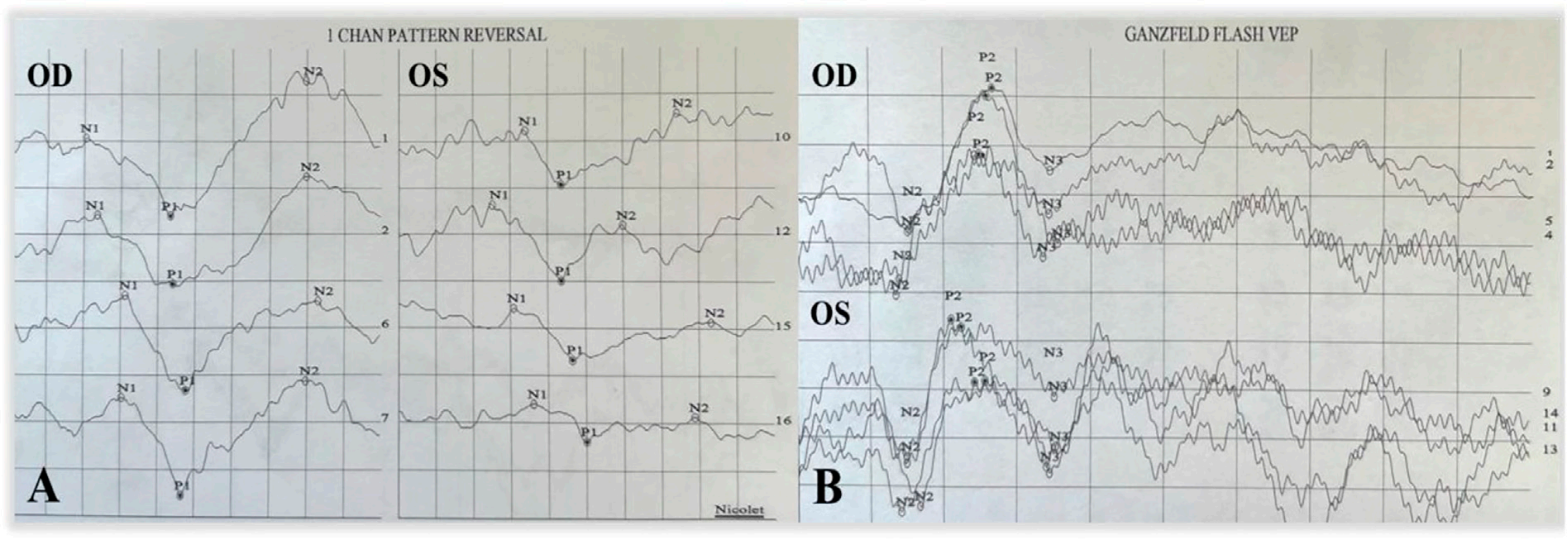
P-VEP and F-VEP of the proband. The latency of the waveform recorded by P-VEP in both eyes was delayed at high SF, which was obvious in the right eye, and the amplitude decreased at high SF, mild in the left eye, and obvious in the right eye **(A)**. The amplitude of the waveform recorded by F-VEP was similar in both eyes **(B)**.

A 9-year-old Asian boy presented with a 2-year history of deteriorating visual acuity in the right eye. He was a full-term fraternal twin, with no oxygen inhalation or other relevant medical history. His best-corrected visual acuity (BCVA) was 7.00/1.75 × 100 = 0.3 in the right eye and 2.50/1.50 × 170 = 0.8 in the left. Intraocular pressure was normal in both eyes. His right eye exhibited exohypertropia with no obvious abnormalities on a slit-lamp examination. Fundus examination revealed that the color of the optic disc in both eyes was slightly pale, the retinal blood vessels were stretched to the temporal side, and the peripheral retinal blood vessels were flat and numerous. A mild stretch proliferation and pigmentation changes were observed in the peripheral retina of the left eye (Figure 1A–B, E–F). Optical coherence tomography (OCT) revealed a diminished foveal contour, with persistent inner foveal retinal layers in both eyes, and no foveal depression on the left eye (Figures 1C,D). Fundus fluorescein angiography (FFA) revealed a large avascular zone in the bilateral temporal peripheral retina, high fluorescence in the optic disc in the late stage of angiography, and patchy high fluorescence with fluorescein staining in the temporal peripheral retina of the left eye (Figure 2). After correcting for refractive error, visual evoked potential (VEP) was performed. The latency of the waveform recorded by the pattern VEP (P-VEP) was delayed and decreased at a high spatial frequency (SF) in both eyes, especially in the right, and there was no significant decrease in the flash VEP (F-VEP) (Figure 3). However, owing to the young age of the patient and unstable fixation, clinical significance should be combined with the overall analysis of various examinations.

**FIGURE 4:**
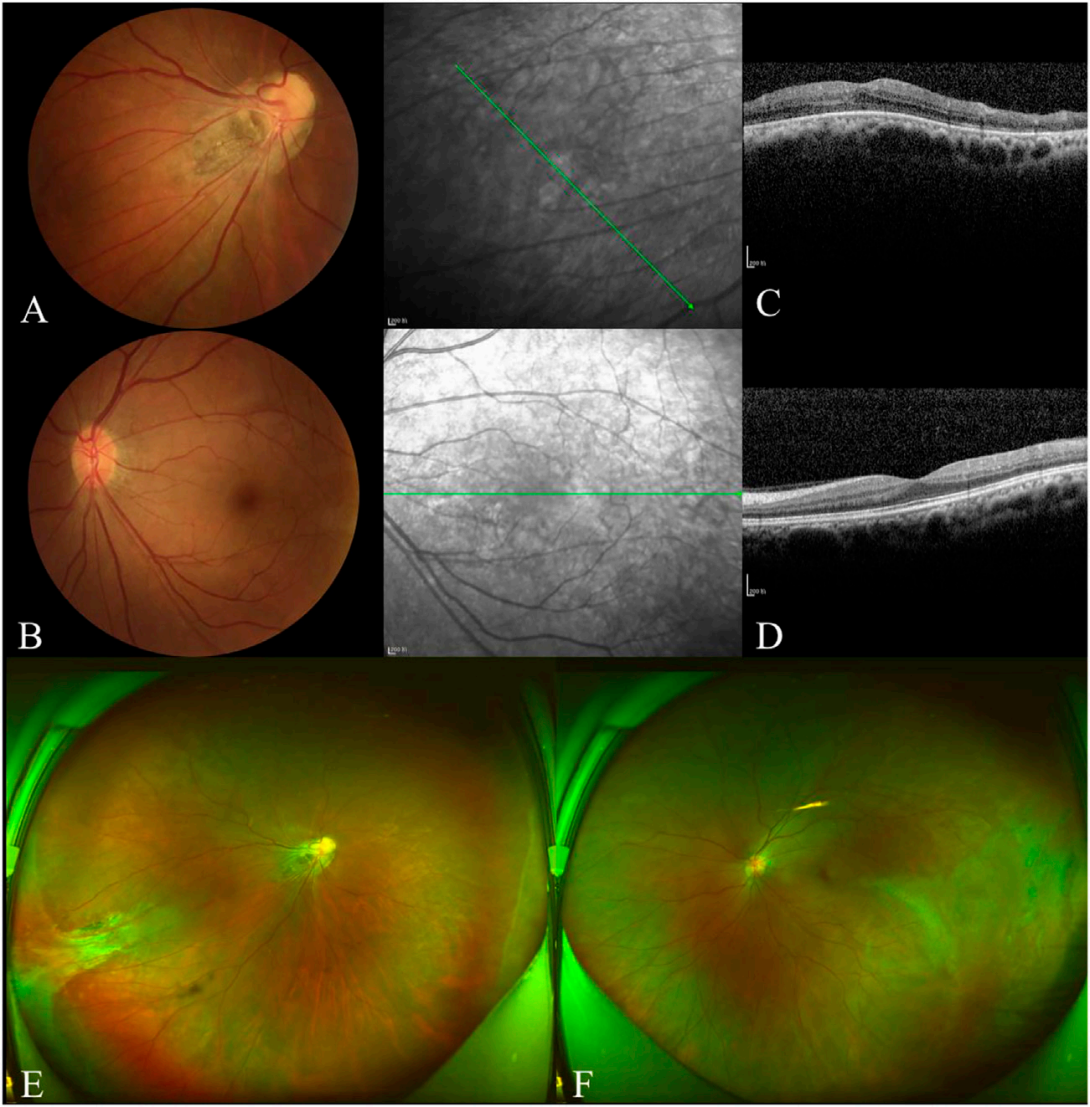
Examination results of the proband’s father. The boundary of the optic disc in the right eye was unclear, and the color was slightly pale **(A)**. The retinal blood vessels were pulled to the temporal side on the right and slightly on the left **(B)**. OCT showed persistent inner foveal retinal layers and diminished foveal contour with IS/OS thinned and discontinuous in the right **(C)**. The structures of the central fovea of the left eye were normal **(D)**. The peripheral retinal blood vessels were flat and numerous in both eyes, accompanied by fiber proliferation and traction, presented as ‘sickle fold’ in the right **(E)**. The peripheral retinal vessels are straight and numerous, accompanied by avascular areas that showed ‘inverted V’ **(F)**.

**FIGURE 5:**
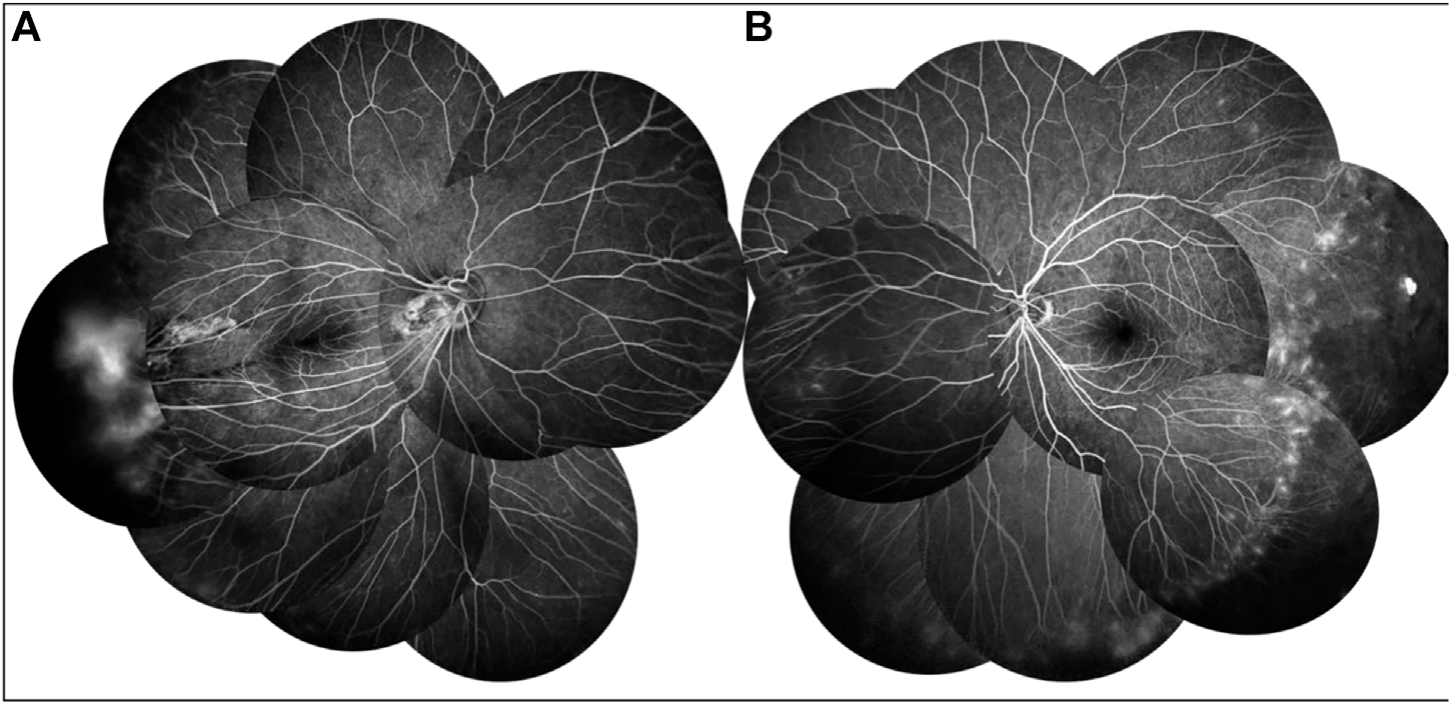
FFA of proband’s father showed a large avascular zone in the bilateral temporal peripheral retina, and blood vessels in the peripheral retinal were flat and numerous **(A,B)**. The temporal peripheral retina showed patchy high fluorescence with fluorescein leakage, which is obvious in the right **(B)**.

The proband’s father was 35 years of age and had a history of left exotropia for the past 35 years. His BCVA was 1.00/2.25 × 75 = 0.05 in the right eye and 0.75 × 75 = 1.0 in the left. His left eye exhibited exotropia and no obvious abnormalities on a slit-lamp examination. Fundus examination revealed that the boundary of the optic disc in the right eye was unclear, and the color was slightly pale. The retinal blood vessels were pulled to the temporal side, and the peripheral retinal blood vessels were flat and numerous, accompanied by fiber proliferation and traction, presenting as a “falciform fold”. The optic disc of the left eye was normal, and the peripheral retinal vessels were straight and numerous, accompanied by avascular areas (Figure 4A, B, E, F). OCT revealed persistent inner foveal retinal layers and diminished foveal contour with IS/OS thinning and discontinuity in the right eye and normal structures of the central fovea in the left eye (Figures 4C,D). FFA revealed a large bilateral avascular zone and patchy high fluorescence with obvious fluorescein leakage in the late stage in the right temporal peripheral retina (Figure 5).

**FIGURE 6:**
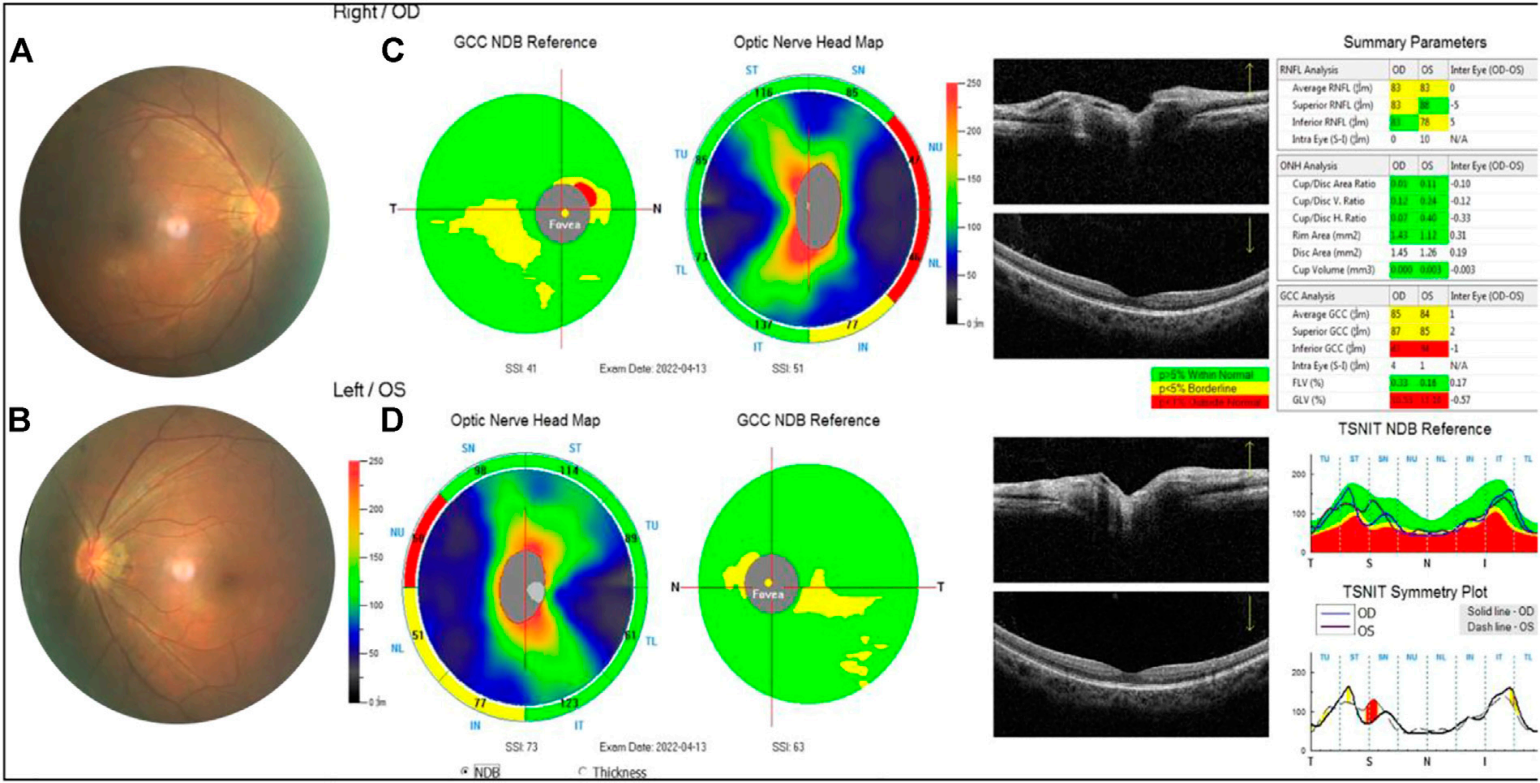
Examination results of the proband’s mother. **(A,B)** Arc spot of myopia in temporal optic discs. **(C,D)** OCT showed that the inner and outer layers of central fovea were normal in both eyes. Optic nerve head analysis was normal. The thickness of inferior RNFL was normal, superior RNFL, and average RNFL was borderline in the right. The thickness of superior RNFL was normal, inferior RNFL, and average RNFL was borderline on the left. The thickness of superior GCC and average GCC was within normal range, and inferior GCC decreased. The focal loss volume (FLV) was within the normal range, and global loss volume was outside the normal range in both eyes.

**FIGURE 7:**
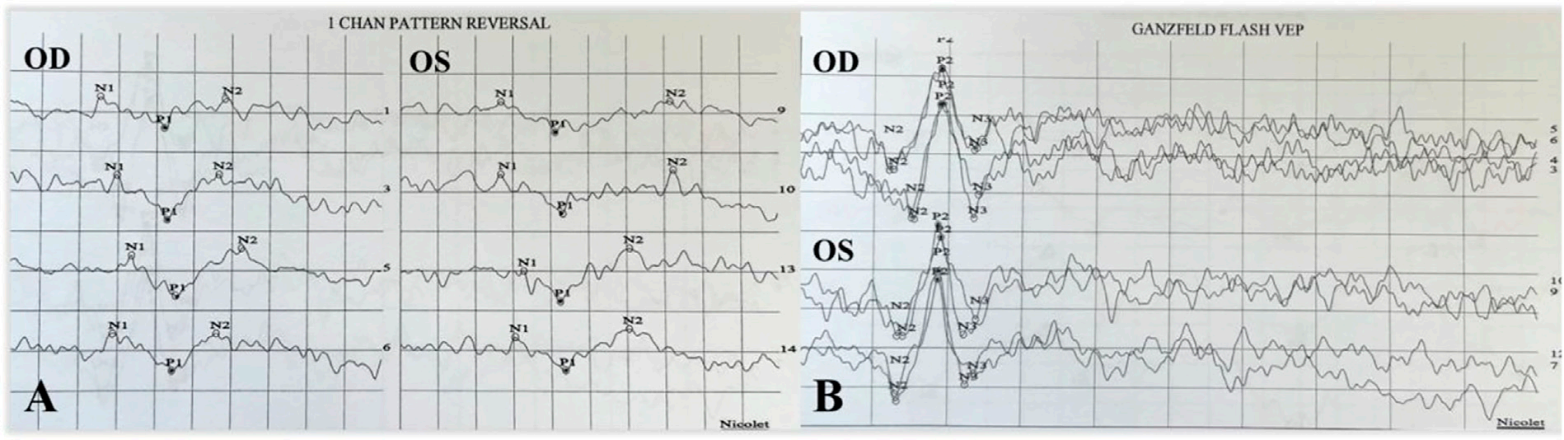
P-VEP and F-VEP of proband’s mother. The latency of the waveform recorded by P-VEP was delayed and decreased slightly at high SF in both eyes **(A)**. The amplitude of the waveform recorded by F-VEP was normal in both eyes **(B)**.

The proband’s mother was 35 years of age, with a history of high myopia, 7.0 diopters oculus uterque (OU), corrected by an implantable collamer lens 10 years ago. Her BCVA was 1.0 in both eyes. Slit-lamp biomicroscopy revealed that the cornea, lens, vitreous, and retina were normal in both eyes. OCT revealed no abnormality in the bilateral central foveae but was thin in the bilateral inferior ganglion cell complex layers (Figure 6). VEP results were within the normal range (Figure 7).

**FIGURE 8:**
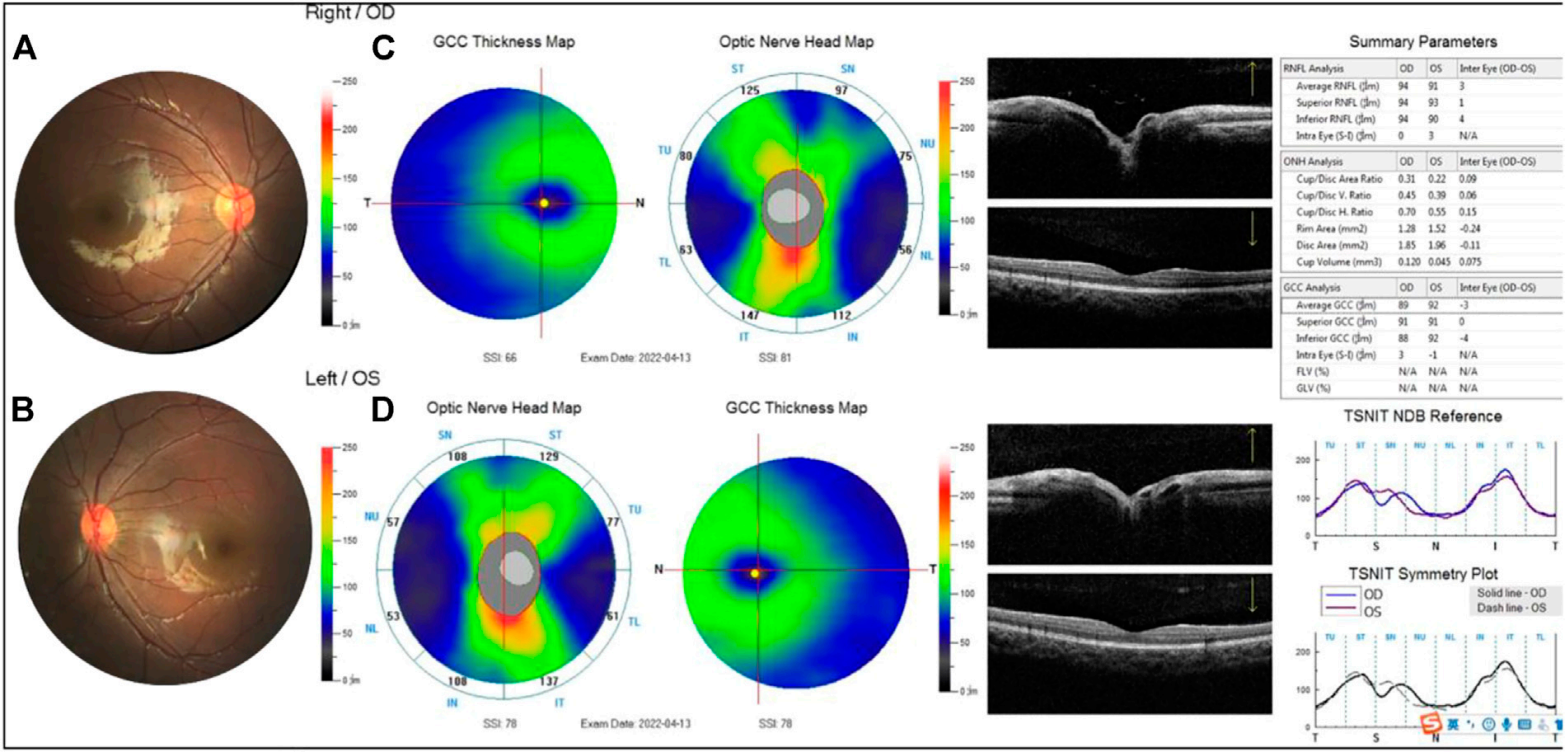
Examination results of the proband’s fraternal twin brother were normal **(A,B)**. The thickness of the superior retinal nerve fiber layer (RNFL), inferior RNFL, and average RNFL was within the normal range. The thickness of the superior ganglion cell complex (GCC), inferior GCC, and average GCC was within the normal range **(C,D)**.

The proband’s fraternal twin brother was 9 years of age and born at full term with no history of oxygen inhalation. His BCVA was 1.0 in both eyes. Slit-lamp biomicroscopy and OCT results were normal in both eyes (Figure 8).

Peripheral venous blood (2 ml) from the patient, his parents, and his brother was collected in the outpatient clinic. Deoxyribonucleic acid (DNA) was extracted using a commercially available blood genomic DNA isolation kit (QIAGEN, Hilden, Germany). The whole exon group of the extracted DNA was detected using a second-generation sequencing technology, and Sanger verification was performed on all subjects (Euler Genomics). The pathogenicity of new mutations was evaluated according to the standards and guidelines for the interpretation of sequence variants issued by the American College of Medical Genetics and Genomics (ACMG) in 2015. With reference to the allele frequencies of East Asians in the 1000 Genomes Project (1000G, http://browser.1000genomes.org) and Exome Aggregation Consortium (ExAC, http://exac.broadinstitute.org/) databases, a minimum allele frequency of <0.005 was defined as the criterion for excluding benign variation(s).

**FIGURE 9:**
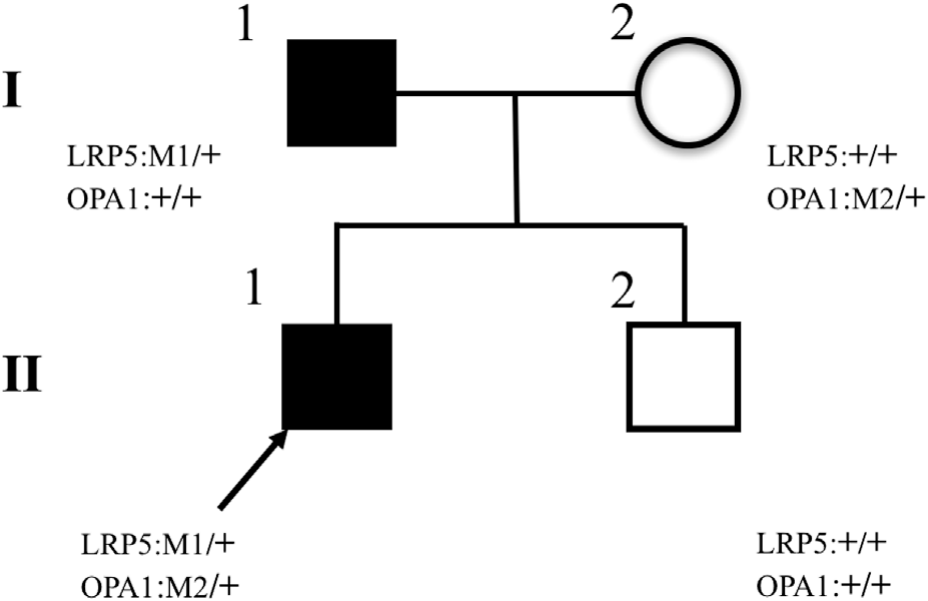
Pedigrees of families. The II–1 proband is marked with an arrow and has two missense heterozygous mutations, LRP5 (c.2551C > T, *p*.His851Tyr) and OPA1 (c.565G > A, *p*.Glu189Lys). Pedigree verification analysis shows that the proband’s father has a heterozygous mutation of LRP5 (c.2551C > T, *p*.His851Tyr) (I–1), the proband’s mother has a heterozygous mutation of OPA1(c.565G > A, *p*.Glu189Lys) (I–2), and the proband’s fraternal twin brother has no mutation (II–2).

**FIGURE 10:**
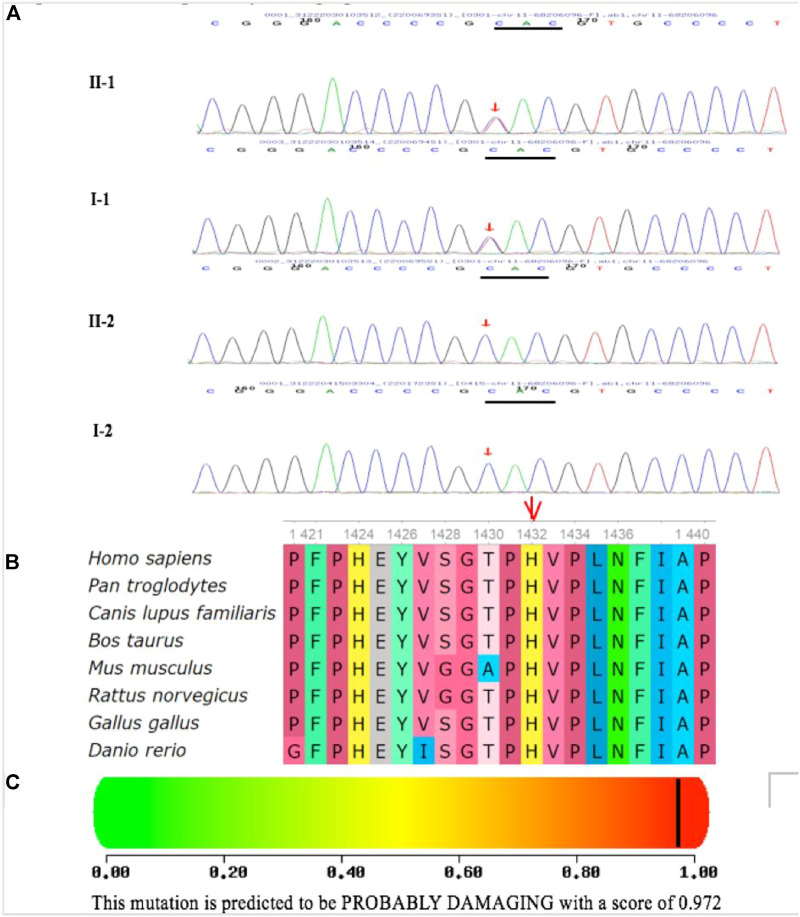
Identification of one heterozygous LRP5 mutation in the family members. **(A)** DNA sequence chromatograms of the unaffected and affected family members. One heterozygous LRP5 mutation c.4294C > T (*p*.H1432Y) in exon 20 was identified in the cases of the proband and his father. **(B)** Multiple-sequence alignment of the LRP5 proteins from different species. The red triangle indicates the location of the mutation. The His 1432 residue is highly conserved across species. **(C)** Polymorphism Phenotyping predicted that the amino acid substitution H1432Y in the protein LRP5 is probably damaging.

**FIGURE 11:**
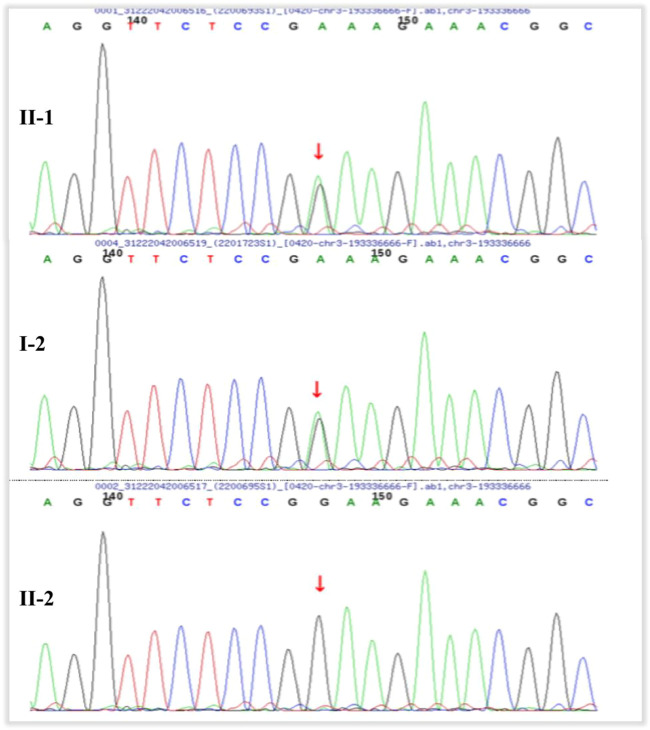
Pedigrees of the families. The proband (II–1) and his mother (I–2) have heterozygous mutation of OPA1 (c.565G > A, *p*.Glu189Lys). The proband’s fraternal twin brother (II–2) has no mutation.

The inheritance mode of this family was autosomal-dominant; the pedigree of the family is presented in Figure 9. Two abnormal genes, *LRP5* and *OPA1*, were detected. The missense mutation in *LRP5* (c. 2551C > T, *p*. His851Tyr) in the proband and his father was a new mutation site, which has not been reported previously; both father and son have normal bone mineral density. Functional prediction revealed that the mutation was likely pathogenic (Figure 10; Table 1). A missense mutation of *OPA1* (c.565G > A, *p*.Glu189Lys) in the proband and his mother has been reported in the literature (Wang et al., 2019; Xu et al., 2021), and its functional prediction was non-pathogenic (Figure 11; Table 1). The patient’s fraternal twin brother did not harbor any of these two abnormal mutations.

**TABLE 1:** Effect of LRP5/OPA1 mutation on protein function by different software applications.

Gene	Software	Nucleotide change	Cite score	Pathogenicity prediction
*LRP5*	Polyphen2_HDIV	c.2551C > T	0.972	Probably_damaging
*LRP5*	SIFT	c.2551C > T	0.63	Tolerable
*LRP5*	PROVEAN	c.2551C > T	−1.54	Tolerable
*LRP5*	MutationTaster	c.2551C > T	0.999	Disease_causing
*OPA1*	Polyphen2_HDIV	c.565G > A	0.039	Benign
*OPA1*	SIFT	c.565G > A	0.92	Tolerable
*OPA1*	PROVEAN	c.565G > A	0.09	Tolerable
*OPA1*	MutationTaster	c.565G > A	1	Polymorphism

## Discussion

FEVR is an inherited developmental disorder caused by incomplete retinal angiogenesis (Criswick and Schepens, 1969). The clinical manifestations of FEVR vary. Some patients can be asymptomatic, even without vascular abnormalities in the peripheral retina, while others can develop retinal detachment and blindness, while still others experience manifestations of varying degrees/severity in between. Pendergast and Trese (1998) proposed a comprehensive systematic classification of the disease. Aside from its phenotypic variability, FEVR is genetically heterogeneous and exhibits dominant, recessive, and X-linked modes of inheritance. To date, mutations in 12 genes have been reported to cause FEVR (Zhu et al., 2021). In addition, FEVR has been linked to mutations in several genes associated with the inactivation of the norrin/β-catenin signaling pathway, accounting for approximately 50% of the cases (Li et al., 2018; Zhu et al., 2021). Among them, the vast majority (90%) of subjects with a molecular diagnosis harbored mutations in four genes: *NDP, FZD4, LRP5*, and *TSPAN12* (Salvo et al., 2015).

The mutation c.2551C > T (*p*.His851Tyr) in exon 12 of *LRP5*, identified in this family, is novel. Compared with *FZD4, TSPN12, NDP*, and *ZNF408*, *LRP5* is most frequently mutated in FEVR, likely due to its large coding region (Molday, 1998; Yang et al., 2012; Musada et al., 2016; Pefkianaki et al., 2016; Li et al., 2017). *LRP5* and its homolog *LRP6* encode a single-span transmembrane receptor, and LRP5/LRP6 interacts with a seven-pass transmembrane receptor. FZD4 and LRP5 form a complex with TSPAN12 in the plasma membrane of endothelial cells (ECs) (Tamai et al., 2000; Xu et al., 2004; Junge et al., 2009; Li et al., 2012). Norrin is an extracellular ligand that binds to FZD4 on ECs with the aid of LRP5 and TSPAN12 to activate downstream β-catenin/Wnt signaling (Tamai et al., 2000; Xu et al., 2004; Junge et al., 2009; Li et al., 2012). Therefore, mutations in any of the genes encoding these five proteins (NDP, FZD4, LRP5, TSPAN12, and β-catenin) may lead to FEVR by inactivating the norrin/β-catenin pathway and altering the expression of its downstream target genes. Although the binding sites of LRP5 to FZD4 and norrin have not been fully characterized, it has been proposed through comparative modeling that missense mutations in *LRP5* may disrupt protein-binding sites (Toomes et al., 2004). Multiple-sequence alignment of the LRP5 protein from different species revealed that the His1432 residue is highly conserved across species. Polymorphism phenotyping predicted that the amino acid substitution H1432Y in LRP5 may be destructive. This domain is responsible for the binding of LRP5/LRP6 to extracellular ligands. Therefore, this mutation may compromise Wnt–β-catenin signaling, which is essential for retinal vascular development. At the same time, patients with LRP5 mutations often have low bone mineral density or a history of fractures (Thomas et al., 2011). The main pathogenesis may be that the LRP5 gene not only participates in retinal vascular development but also plays a role in promoting osteoblasts and bone formation by inhibiting serotonin synthesis in chromaffin-like cells.

The proband and his father had FEVR in both eyes, and a new missense heterozygous mutation site on *LRP5* was detected. Neither the father nor the son has abnormal bone mineral density. According to the ACMG guidelines, combined with the accumulation of evidence from functional assays, software predictions, and functional domains, the pathogenic possibility of this site was determined, which is consistent with the phenomenon of family co-segregation. According to Pendergast and Trese (1998), the stages of FEVR in the proband were stage 1 in the right eye and stage 2 in the left eye, while his father was at stage 3 in the right eye and stage 1 in the left eye. OCT revealed central foveal hypoplasia in both eyes of the proband and, according to the classification proposed by Mervyn et al. (Yonekawa et al., 2015), grade 1 in the right eye, grade 2 in the left eye of the proband, and grade 2 with IS/OS thinning and discontinuity in the right eye of his father. Previous studies (Barboni et al., 2014) have reported that persistence of the inner retinal layers in the fovea was observed in 20% of patients with stages 1 and 2 FEVR. This single developmental abnormality does not appear obvious if the outer retinal structures remain intact. The left eye of patients had a higher grade of central foveal hypoplasia on OCT; however, BCVA reached 0.8, while that of the right eye was only 0.3, indicating that poor vision in the right eye was not due to central foveal hypoplasia related to FEVR. Similar to many other macular pathologies, atrophy of the outer retinal structures, such as the external limiting membrane, ellipsoid zone, and interdigitation zone, was the strongest independent risk factor for poor visual acuity in eyes with FEVR (Barboni et al., 2014). This could explain why BCVA in the right eye of the proband’s father was only 0.05 because the inner layers did not develop well, and the IS/OS was injured in the fovea. Because FEVR is not a primary degeneration of photoreceptors, these outer retinal changes are likely secondary to damage from the overlying intraretinal and vitreoretinal interface pathology.

In the present study, the proband harbored gene mutations that could cause two different diseases. To the best of our knowledge, c.565G > A *p*.Glu189Lys in *OPA1* has not been reported in patients with FEVR. The genotype–phenotype correlation is weak, with significant variability in both penetrance and clinical severity. *OPA1* encodes a dynamin-related GTPase. OCT can reveal diffuse thinning of the ganglion cell complex layers and retinal nerve fiber layers on the temporal side of the optic disc in patients with ADOA (Barboni et al., 2014; Pretegiani et al., 2017; Ham et al., 2019). Although the proband and his mother harbored mutations in *OPA1*, ADOA could be clinically excluded through fundus, OCT, and VEP examination results. In addition, bioinformatics analysis by Polymorphism Phenotyping (i.e., PolyPhen) and Sorting Intolerant From Tolerant (i.e., SIFT) predicted that the mutation c.565G > A *p*.Glu189Lys in *OPA1* was harmless, and it has been reported to be uncertain in the existing literature (Wang et al., 2019; Xu et al., 2021). Therefore, the proband still has the possibility of developing ADOA in the future and, as such, requires long-term observation. Combined with the clinical manifestations of the proband and the results of multiple examinations, the reason for the poor vision in the right eye was monocular amblyopia caused by high myopia and anisometropia.

## Conclusion

This case report expands the *LRP5* gene mutation spectrum among Chinese patients with FEVR and reports a patient harboring both LRP5 and OPA1 gene mutations to have anisometropic amblyopia and strabismus as the primary manifestations for the first time, which provides clinical clues for a more comprehensive understanding of the disease and will be valuable for genetic counseling and development of therapeutic interventions for patients with FEVR. Because FEVR is a clinically heterogeneous disease, molecular diagnosis provides useful information for disease diagnosis and genetic counseling. Although our understanding of the function of LRP5 and OPA1 proteins remains limited, the discovery of these mutant variants provides an opportunity and rationale for in-depth mechanistic studies and may help reveal the critical physiology underlying retinal development disorders in general. These four family members exhibited individual heterogeneity of phenotypes and genotypes associated with hereditary ophthalmopathy.

## Data Availability

The datasets for this article are not publicly available due to concerns regarding participant/patient anonymity. Requests to access the datasets should be directed to the corresponding author.

## Abbreviations

FEVR, familial exudative vitreoretinopathy; ADOA, autosomal dominant optic atrophy; DNA, deoxyribonucleic acid; ACMG, American College of Medical Genetics and Genomics; ExAC, Exome Aggregation Consortium; BCVA, best-corrected visual acuity; OCT, optical coherence tomography; FFA, fundus fluorescein angiography; VEP, visual evoked potential; SF, spatial frequency; P-VEP, pattern visual evoked potential; F-VEP, flash visual evoked potential; OU, oculus uterque; ECs, endothelial cells ; PolyPhen, Polymorphism Phenotyping; SIFT, Sorting Intolerant From Tolerant.
